# Supplementary material for: Synthesis, Characterization, and Application of Dichloride (5,10,15,20-Tetraphenylporphyrinato) Antimony Functionalized Pectin Biopolymer to Methylene Blue Adsorption
Source: Polymers (Basel). 2023 Feb 19;15(4):1030. doi: 10.3390/polym15041030 (PMC9968078; doi:10.3390/polym15041030)
Supplement: Supplementary file 1 [file polymers-15-01030-s001.zip › polymers-2136242-supplementary.pdf]

polymers

MDPI

Supplementary File

Synthesis, Characterization, and Application of Dichloride (5,10,15,20-Tetraphenylporphyrinato) Antimony Functionalized Pectin Biopolymer to Methylene Blue Adsorption

Raoudha Soury [1,*], Munirah Sulaiman Othman Alhar [1] and Mahjoub Jabli [2,*]

1 Department of Chemistry, College of Science, University of Hail, Ha'il 81451, Saudi Arabia
2 Department of Chemistry, College of Science Al-Zulfi, Majmaah University, Al-Majmaah 11952, Saudi Arabia
* Correspondence: ra.soury@uoh.edu.sa (R.S.); m.jabli@mu.edu.sa (M.J.)

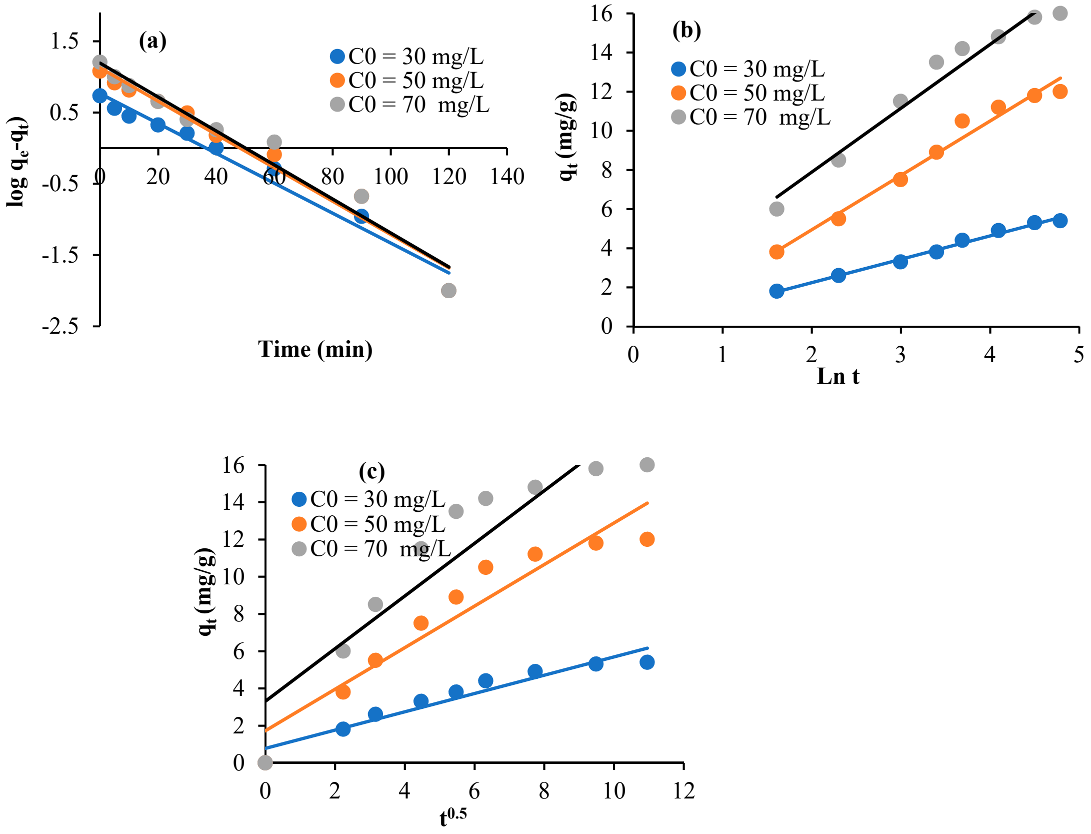

Figure S1. Kinetic plots for pectin beads: **(a)** First order, **(b)** Elovich, and **(c)** Intra-particular diffusion.

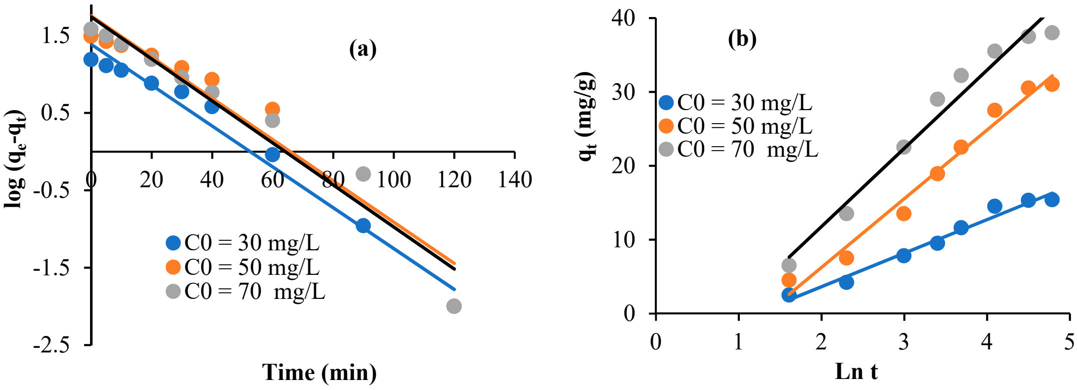

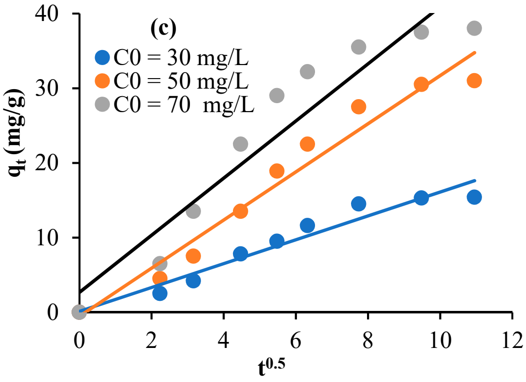

Figure S2. Kinetic plots for pectin-[Sb(TPP)Cl2] beads (1%): **(a)** First order, **(b)** Elovich, and **(c)** Intra-particular diffusion.

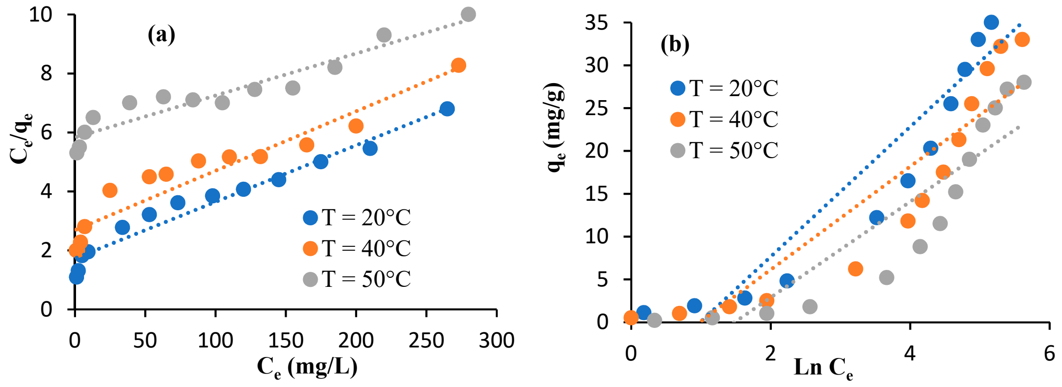

Figure S3. Isotherm data modeling for pectin beads: (a) Langmuir, and (**b**) Temkin.

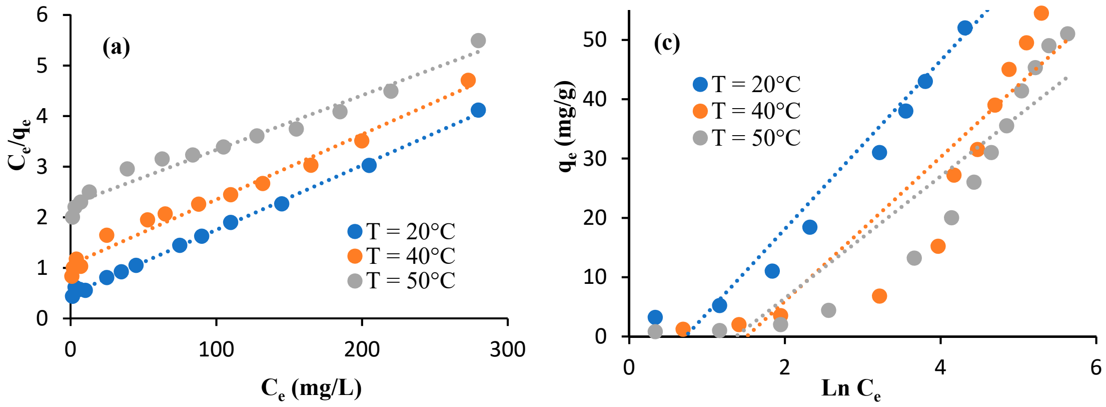

Figure S4. Isotherm data modeling for pectin-[Sb(TPP)Cl2] beads (1%): (a) Langmuir, and (**b**) Temkin.
